# PathoGraph: An Attention-Based Graph Neural Network Capable of Prognostication Based on CD276 Labelling of Malignant Glioma Cells

**DOI:** 10.3390/cancers16040750

**Published:** 2024-02-11

**Authors:** Islam Alzoubi, Lin Zhang, Yuqi Zheng, Christina Loh, Xiuying Wang, Manuel B. Graeber

**Affiliations:** 1 School of Computer Science, The University of Sydney, J12/1 Cleveland St, Darlington, Sydney, NSW 2008, Australia; ialz9547@uni.sydney.edu.au (I.A.); lzha8455@uni.sydney.edu.au (L.Z.); 2 Ken Parker Brain Tumour Research Laboratories, Brain and Mind Centre, Faculty of Medicine and Health, University of Sydney, Camperdown, NSW 2050, Australia; yzhe8012@gmail.com (Y.Z.); christina14@protonmail.ch (C.L.); 3 University of Sydney Association of Professors (USAP), University of Sydney, Sydney, NSW 2006, Australia

**Keywords:** artificial intelligence, attention mechanism, CD276, graph neural network (GNN), histological analysis

## Abstract

**Simple Summary:**

We have developed a graph- and attention-based A.I. model (PathoGraph) that predicts survival and identifies tissue areas of special diagnostic interest in whole slide images (WSI) of glioblastoma. CD276, an important immune checkpoint molecule, was confirmed to be a marker of malignant glioma cells/putative glioma stem cells (GSCs). The previously developed PathoFusion framework was used to selectively detect these cells. The graphs and attention scores that were obtained on the basis of this selection provide information that is hidden from conventional microscopic observation.

**Abstract:**

Computerized methods have been developed that allow quantitative morphological analyses of whole slide images (WSIs), e.g., of immunohistochemical stains. The latter are attractive because they can provide high-resolution data on the distribution of proteins in tissue. However, many immunohistochemical results are complex because the protein of interest occurs in multiple locations (in different cells and also extracellularly). We have recently established an artificial intelligence framework, PathoFusion which utilises a bifocal convolutional neural network (BCNN) model for detecting and counting arbitrarily definable morphological structures. We have now complemented this model by adding an attention-based graph neural network (abGCN) for the advanced analysis and automated interpretation of such data. Classical convolutional neural network (CNN) models suffer from limitations when handling global information. In contrast, our abGCN is capable of creating a graph representation of cellular detail from entire WSIs. This abGCN method combines attention learning with visualisation techniques that pinpoint the location of informative cells and highlight cell–cell interactions. We have analysed cellular labelling for CD276, a protein of great interest in cancer immunology and a potential marker of malignant glioma cells/putative glioma stem cells (GSCs). We are especially interested in the relationship between CD276 expression and prognosis. The graphs permit predicting individual patient survival on the basis of GSC community features. Our experiments lay a foundation for the use of the BCNN-abGCN tool chain in automated diagnostic prognostication using immunohistochemically labelled histological slides, but the method is essentially generic and potentially a widely usable tool in medical research and AI based healthcare applications.

## 1. Introduction

In recent years, the clinical practice of pathology has been advanced by the introduction of slide scanners for the generation of multi-resolution WSIs. With the arrival of digital pathology, computer-assisted diagnostic (CAD) algorithms have begun to develop traction. CAD systems are now capable of supporting the diagnostic work-flow in pathology, improving efficiency and facilitating decision making [1,2]. WSIs used at high resolution can be extremely large, often including billions of pixels, making them computationally intensive to process and analyse [3]. In addition to image size, the complexity of tissue architecture and variability in immunolabelling intensity contribute to the difficulties of computational WSI analysis. Many existing methods rely on manual or semi-manual annotating of regions of interest (ROI), which is subject to inter-observer variation and can be very time-consuming.

Deep learning approaches are showing promise in achieving high levels of accuracy in image analysis tasks, they allow data reduction, and enable feature selection techniques that increase computational efficiency [4,5,6,7,8,9]. Deep learning algorithms, particularly convolutional neural networks (CNN), are able to extract morphological features automatically after appropriate training [10], and many researchers in the field have adopted a patch-based approach to extract features while preserving essential information for detection and classification tasks [11,12,13,14,15].

CNNs are designed to operate well on local portions of an image, e.g., extracting features from ROIs using convolutional filters. While this method is effective for capturing local patterns and features, it is not well suited for capturing a WSI’s global context and structure [16]. Graph-based deep learning methods have been proposed to bridge this gap and to model the geometric structure of the tissue at both local and global levels [17,18].

Cell graph neural networks (CGNNs) are a type of GNN where a graph is constructed using the specific locations of cells or cell nuclei as entities or “nodes” while the spatial distances between them [19,20] are represented as lines which are called “edges”. Attention mechanisms enable the network to focus on specific ROIs in an image resulting in improved accuracy of output. GNNs and attention mechanisms can also be used together to further improve the performance in image analysis tasks [21].

**Figure 1:**
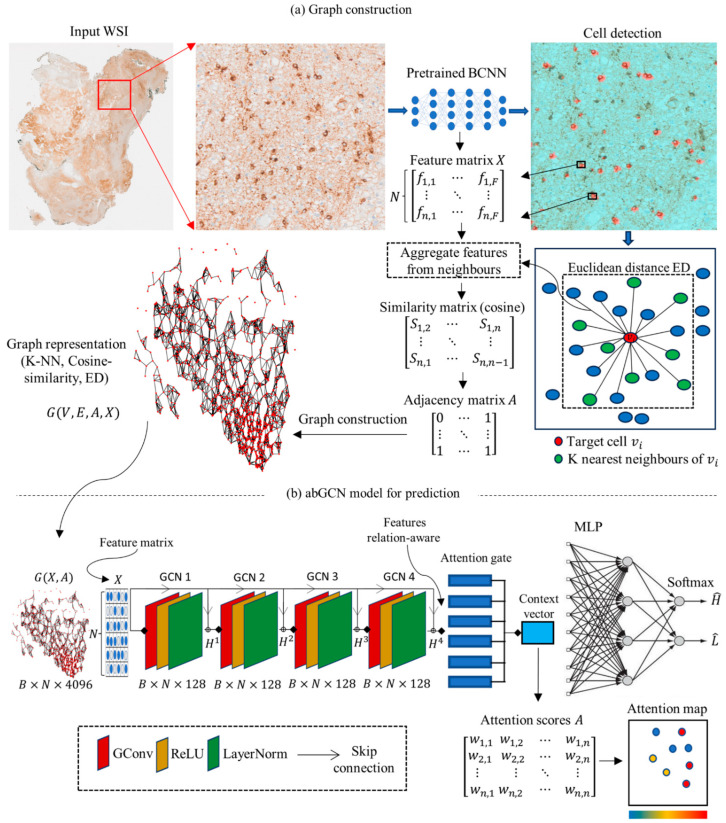
PathoGraph framework. (**a**) Graph construction. The previously developed BCNN model (PathoFusion) is used to identify CD276 immunoreactive GSCs, extract their centroids as vertices of a graph and their features as vertex features X. Edge features are captured considering both spatial distance and the feature cosine similarity between neighbouring cells following mapping of Euclidean distance, ED. The constructed graphs are visualized using pseudo-colours illustrating the connection between the detected cells across the entire tissue section. (**b**) AbGCN model for prediction. The constructed graphs are then fed into our abGCN model that has undergone supervised training on a training cohort of cases to predict survival probabilities. The attention scores are converted into attention maps (see Figure 5).

We have recently explored the capability of our bifocal convolutional neural network (BCNN) model [22], the core of the PathoFusion framework [23], to identify immunolabeled cells and characterise pathological changes at the cellular level [1]. The current study extends this work by adding a comprehensive GNN framework, termed PathoGraph, which utilises attention mechanisms and models cell level information and overall tissue micro-architecture. This approach is based on the assumption that cells in a tissue can organize in a certain way (e.g., clones of cancer cells) which may relate to prognosis and ultimately survival. Specifically, we introduce a model which constructs a graph beginning at the cellular level to include the entire WSI (Figure 1). A graph convolutional neural network (GCN) with attention mechanisms (abGCN) is then used for WSI-level survival prediction and to pinpoint cell populations that deserve the greatest attention.

The example used in this study is CD276 expression by malignant glioma cells/putative glioma stem cells (GSCs). CD276, also known as B7-H3, serves as a key immune checkpoint molecule which is highly expressed in malignant tumours. Its expression is thought to exert an inhibitory effect on the activation and proliferation of T cells [24]. Upregulated expression of B7-H3 in various cancers, including acute leukaemia, ovarian cancer and GBMs [25], has been associated with poor prognosis [26,27]. In this study, we are focusing on the strongly CD276 immunoreactive “halo” cells described earlier [1].

## 2. Materials and Methods

### 2.1. Dataset 

We conducted experiments on two datasets to validate the effectiveness of our model. One imaging dataset was as before [1]. We have used 32 WSIs of immunohistochemically stained tissue sections with an average size of 100,225 × 150,537 pixels at 40× primary magnification. As described [1], immunohistochemistry for CD276 was carried out on an automated Ventana system. The SP265 antibody (Abcam) directed against C-terminal CD276 was used [1]. Immunoreactive cells were individually marked on digital slide scans using the PathoFusion platform which integrates a labelling website [23] under supervision of a consultant neuropathologist, resulting in the placement of a total of 50,741 marking dots over glioma cells showing strong CD276 expression. They were used for training the BCNN model [22]. A second dataset was obtained from TCGA. This glioblastoma (GBM) dataset contains 255 WSIs [28] and was used to assess our model’s effectiveness.

### 2.2. The Framework

PathoGraph is modelled after a diagnostic workflow, i.e., it follows principles that aid pathologists when they evaluate histopathological slides [23]. Accordingly, high resolution features such as cellular morphology, cellular position, and the expression of certain molecular markers, as well as global features such as anatomical information and landmarks, tissue layers, cellular (community) patterns, and overall tissue organization are taken into account. Figure 1 shows an overview of the PathoGraph framework. For any given WSI, we used our pre-trained BCNN model [1] to detect CD276 immunoreactive cells. We then used their positions as vertices as well as their features, which were extracted using the same model. Edges were captured taking into account both spatial relationships and feature similarities between neighbouring cells based on a given Euclidean distance (ED). The constructed graphs were then visualized illustrating the connection between the detected cells across each tissue section. Each tissue image was analysed by feeding its graph into our abGCN model that had undergone supervised training on our training cohort of cases. The proposed abGCN model enables the learning of a data-driven graph feature hierarchy without the need for hand-crafted features. The attention mechanism in our model allows the network to focus on the most relevant information (important cells) in the graph, making the model more interpretable. The attention scores were finally converted into maps.

### 2.3. Graph Construction on WSI

A pretrained BCNN model was used to detect the CD276-expressing cell population of cells [1]. Once the cells had been detected, neuropathological features were extracted from 64 × 64 patches, each centred at the detection peak location. These features serve as vertex features derived from the first layers of the BCNN model. Then, an average-pooling layer was utilised to generate a 4096-dimensional feature vector. The spatial distance and cosine similarity of the features extracted by the pre-trained BCNN, which describes the similarity between patches, were taken into account. Since WSIs are gigapixels in size, aiming to represent a raw WSI, using all the information available at the single pixel level in a graph is computationally challenging. Therefore, we propose a memory-efficient WSI encoding method that uses a set of subgraphs which represent the gigapixel-sized image in a memory-efficient vector space (Algorithm 1). We build these sub-graphs by iteratively identifying nearby cells based on their centre coordinates and feature characteristics and then draw edges between them based on distance, nearest neighbourhood, and cosine similarity for each WSI as described below. This subgraph approach efficiently reduces a graph’s data size while preserving spatial relationships between patches.

We employed a maximum ED of 500 pixels as an iteratively determined threshold for the automated construction of edges between the most relevant subset of nodes (CD276 immunoreactive cells [1]). This allows a contextual analysis of the cell’s connectivity and its relationship to regions of interest in the WSI defined by pathological features that are recognizable when using other modalities on adjacent sections [23]. The nodes (vertices) that are used have similar features based on cosine similarity. The k− nearest neighbours (k = 8) of each cell that lie within the similarity threshold (cosine similarity) of the patch features which have been extracted from the final multi-layer perceptron (MLP) layer of the pre-trained BCNN model are then determined. Patches are accepted as similar if the value for the cosine similarity is greater than the given threshold. Thus, following the identification of the relevant patches within the WSI, a graph’s edges are created based on spatial distance and feature similarity of the patches. Furthermore, to demonstrate that the proposed method is appropriate for representing the WSI by means of a compressed graph structure, we compared our method to two other graph generation methods, Fully Connected (FC) and Multiple Instance Selection (MIS), respectively. The MIS method randomly samples small patches within a distance threshold independent of the presence of cells of interest whereas the FC graph method includes the extracted cells but constructs one graph without being constrained by limitations on the distances between the connected patches. We found that the proposed graph construction method improves prediction accuracy of the abGCN model significantly compared to the other two methods tested. 

Therefore, for a given WSI, after localizing CD276 immunoreactive cells and extracting their features using our pretrained BCNN model, we constructed a graph G(V,E,A,X). For this, we treated each patch n∈N as a node to construct a set of vertex patches $V=\left\{V_{n}\;\right|\;n\epsilon N\},$ such that each node is represented by the geometric center c  of the patch and embeds the micro-level features of each cell in a patch. The edge set E⊆V×V represents the connections between nodes expressed by a sparse adjacency matrix A, in which ${A\;}_{ij}$ depicts the relationship between node i and node j. WSI graphs were encoded by calculating spatial adjacency and feature similarity between all patches and storing node level embeddings (features) in the attribute matrix X. The resulting graph was then visualized using pseudo-colours illustrating the connections between the detected cells across the entire tissue section.
**Algorithm 1**: Graph construction for WSI**Input**: **WSI #** whole slide image 

**Initialize**: 
V⟸∅
**#** vertex set 
E⟸∅
**#** edge set 
K = 8 # the number of nearest neighbors to connect 
ED=500 pixels # Euclidean distance threshold to determine nearby cells 
CT=0.8
**#**cosine similarity threshold 

**N** = **patches number #** patches including cells detected by BCNN from WSI 
**for n** in **N do**
    $c_{i}=$ geometric center of the patch     $f_{i}=$
**BCNN(n) #**extract features for the patch     **Add**
$v_{i}=\left(c_{i},f_{i}\right)$ to V

**end for**


**for**
$v_{i}$ in V
**do**
    ${Neighbors}_{i}⟸\emptyset$
    $Nearest\;{Neighbors}_{i}⟸\emptyset$

    **EDS** = {Euclidean Distance ($v_{i}$, $v_{j}$)|$v_{j}\epsilon V$ }     **for** ed in **EDS do**
      **if** ed ≤
**ED do**              **CS** = Cosine Similarity ($v_{i}$, $v_{j}$)        **Add** {$v_{j}$, CS} to ${Neighbors}_{i}$
      **end if**
    **end for**

    ${SortedNeighbors}_{i}$ = **Sort** (${Neighbors}_{i}$) # Descending by cosine similarity values     Count = 0     **for** {$v_{j}$, CS} in ${SortedNeighbors}_{i}$
**do**
      **if** Count < K and CS ≥ CT
**do**
       **Add**
$v_{j}$ to $Nearest\;{Neighbors}_{i}$
      **end if**
      Count = Count + 1     **end for**

    **for** nn in $Nearest\;{Neighbors}_{i}$
**do**
      **Add** edge ($v_{i}$, nn) to **E**
    **end for**

**end for**


**Output**:  Graph representation G=(V,E) of the WSI

### 2.4. Graph Convolutional Network

A spatial GCN considers the neighbouring nodes up to a certain distance (n-hop neighbours), unlike spectral GCN graph approaches, which take homogeneous graph datasets as inputs with the adjacency matrix fixed across the dataset. Since tissue images can have greatly variable characteristics, PathoGraph must handle different types of nodes and connectivity patterns.

PathoGraph was developed to relate cell community characteristics to system outcome. In our specific application, prognosis can be inferred from the distribution of strongly CD276 immunoreactive glioma cells that may represent stem cells which are represented by graphs that are then related to existing survival data of the cases by the abGCN model. From a general application point of view, we suggest that both the input parameters and the system outcome that is being determined can be varied quite freely, e.g., to identify and validate new biological markers that are of a higher system level relevance. Our visualisation technique emphasizes tissue areas characterized by high attention scores that appear to match the tissue localization of progressive tumours in neuropathological evaluations but overall, the discriminative power of the abGCN seems much higher when relating the results to survival data.

After constructing the graph on the WSI, a classification algorithm was used to explore biological associations of the data. Given the comparatively small number of WSIs in the first dataset, a dichotomous classification algorithm was used. We found that graphs of greater complexity, as expressed by the number of nodes and edges in each cell community, correlated with shorter survival. We also found that attention scores were a measure of complexity at shorter Euclidian distance (256 pixels).

**Figure 2:**
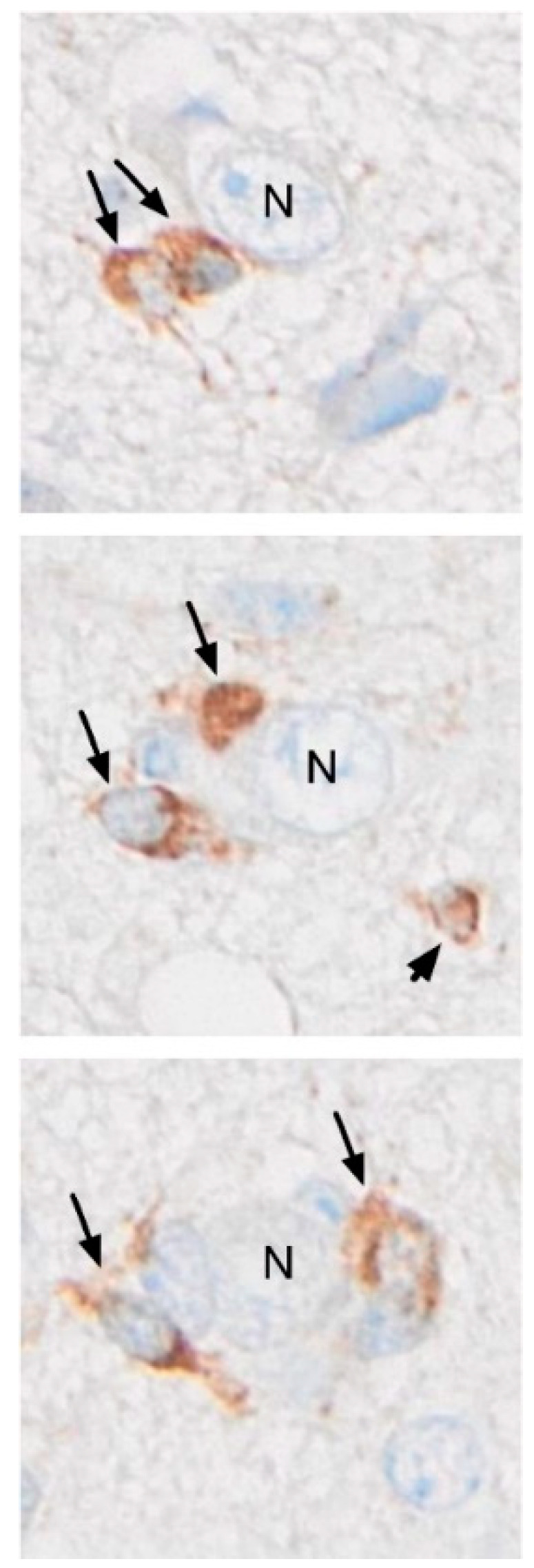
CD276 expression by malignant glioma cells/glioma stem cells (GSCs) in a typical perineuronal satellite position (arrows; N, neuron). CD276+ GSCs are also found perivascularly and accumulating in a subpial location (so-called secondary structuring according to Scherer, not shown). The arrowhead points to a free-lying glioma cell.

Thus, within PathoGraph, the task of predicting disease (system) outcome can be considered a graph classification problem at the WSI-level with a WSI representing the disease substrate contained in a biopsy. We anticipate that the approach can be generalized to employ other laboratory data as well as system outcome parameters. The system outcome we are especially interested in for our example is patient survival as the cell population which expresses CD276 strongly in the cytoplasm includes pathognomonic perineuronal satellite glioma cells (Figure 2).

GCNs rely on message passing, which means that vertices exchange information with their neighbours and send messages to each other. GCNs initiate with a node creating a feature vector that represents the message (feature vector values) it passes on to all its neighbours, so each node receives one message per adjacent node. Messages vary across nodes and embeddings of nodes are updated by their neighbours. The total number of messages each node receives is obtained by the GCN layer which is defined as follows [29]:

(1) $$H^{\left(l+1\right)}=\sigma (D^{-\frac{1}{2}}\overset{´}{A}D^{-\frac{1}{2}}H^{\left(l\right)}W^{\left(l\right)})$$

$H^{l}$ is the node feature matrix at the l−th layer, where each row represents the feature vector of a node, $W^{\left(l\right)}$ is the weight matrix for the l−th layer where the input features are transformed into messages $\left(H^{l}W^{l}\right)$. The identity matrix I was added to the adjacency matrix A so that each node sends its own message also to itself: $\overset{´}{A}=A+I$. Finally, in order to take the average instead of summing, we calculate the matrix D which is a diagonal matrix with $D_{ii}$ denoting the number of neighbours node i has. σ represents a non-linear activation function, a ReLU-based activation function in this case.

We have used the abGCN as our base model (Figure 1). This network consists of a sequence of GCN layers and non-linearities such as ReLU which enables optimised message exchange between nodes and captures more complex patterns and relationships in the graph. Furthermore, to prevent overfitting, we employ LayerNorm followed by ReLU activation and also skip connections (residual connections) between each layer to mitigate the vanishing gradient problem and improve learning capacities and stability. We used one attention gate (32 dimensions) with three layers, which has a receptive field large enough to capture environmental features with the GCN. We use the MLP layer and the last fully connected layer to make the final classification decision as the WSI output. PyTorch and PyTorch Geometric were used for our implementations.

### 2.5. Graph Attention

In a standard GCN layer, filtering is performed by aggregating the features of neighbouring nodes using a shared weight matrix. In contrast, a graph attention layer extends the traditional GCN by incorporating an attention mechanism. This means the filtering operation is enhanced by considering the importance of neighbouring nodes through assigning specific weights to individual inputs [30]. Analysis of the learned weights after applying the attention mechanism to them can provide insights into how the model is making decisions, enhancing interpretability.

Computation in a graph attention layer typically involves computing attention coefficients and weighting features, and aggregating them, which is performed for each node in the graph. Attention coefficients are computed by comparing a node’s features with the features of its neighbours. These coefficients determine the importance of each neighbouring node in relation to the central node. The attention coefficients are then used to give weight to the features of the neighbouring nodes; hence, they emphasize the features of more important neighbours and reduce the weight of features of less important nodes. The weighted neighbour features are then aggregated by summing them up to capture the information from neighbouring nodes that is relevant to the central node. Lastly, the aggregated representation can be passed through a ReLU activation function to make the output non-linear for normalization. We also compute the self-attention score between adjacent neighbourhood patches to take into account the heterogeneity of the surrounding features. Furthermore, the spatial position of each histological feature must be considered. Therefore, we include the distance feature in the attention score calculation so that positional information is passed between the patches.

Taken together, during processing by the PathoGraph framework, each WSI is characterised by feeding its corresponding graph into the abGCN model which has undergone supervised training on a cohort of cases.

## 3. Results

A summary of our new method, PathoGraph, is shown in Figure 1. To assess the PathoGraph framework’s efficacy in predicting system outcome, survival in this case, we have visualized graphs generated from immunohistochemically labelled histopathological image scans, compared the prediction performance of our abGCN model with state-of-the-art architectures, and visualized GSCs bearing high attention scores in tissue.

We have used two datasets for our study, our own CD276 dataset [1] and glioblastoma (GBM) dataset from TCGA [28]. Our CD276 dataset [1] consists of high-grade glioma cases from the Australian Genomics and Clinical Outcomes of Glioma (AGOG) repository. Almost all are glioblastomas according to the most recent, revised 2021 WHO classification of CNS tumours but one case needed to be reclassified because it carries an IDH1 mutation (R132H). Importantly, this difference in genotype turned out to be irrelevant for our study because the CD276 expression by GSCs in this case was as strong or stronger as in the other, non-IDH1 mutated tumours suggesting that CD276 expression by malignant glioma cells is a fundamental characteristic of these tumours that dominates the effect of an IDH1 mutation.

It is also worth noting that for the first time according to our knowledge, CD276 expression was shown to occur in the typical perineuronal satellite glioma cells (a classical secondary structure according to Scherer) confirming the identity of the CD276 immunoreactive cells as malignant glioma cells/GSCs and emphasizing the importance of the immune checkpoint-marker, CD276, for the biology of diffuse malignant glioma (Figure 2).

### 3.1. Correlation between CD276 Immunoreactive Cell Communities and Survival

One aspect of this work focuses on the evaluation of the relationship between the presence of CD276 immunoreactive cells and patient survival in glioblastoma as an example of system outcome predicted from WSI graph data. In our previous work, we have demonstrated the ability of our BCNN model to detect and identify cells of interest efficiently and consistently in WSIs based on predefined profiles. We now take advantage of the detection capability of our BCNN model to additionally count the relevant cells.

Interestingly, the number of CD276 GSCs in the material available to us varied significantly in line with the histological heterogeneity of glioblastoma and the inherent variability of the neurosurgical bioptic process. This facilitated illustration of the fact that attention scores do not simply reflect cell numbers but are based on graph features. Since the number of CD276 positive cells varied greatly (from less than 100 to 15,000 per WSI), our analysis also shows that the abGCN method we are proposing is capable of dealing with a wide dynamic range.

**Figure 3:**
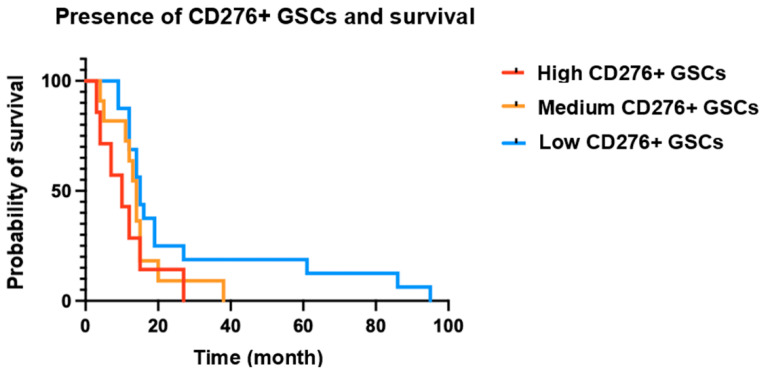
Correlation between the number of CD276 immunoreactive GSCs and patient survival.

We have performed a Kaplan–Meier analysis on our dataset to evaluate the correlation between the number of CD276 immunoreactive GSCs and patient survival (Figure 3) and found that the number of CD276 positive GSCs is associated with patient survival with p=0.05 (Appendix A).

### 3.2. Visualization of the Graphs Constructed by PathoGraph

**Figure 4:**
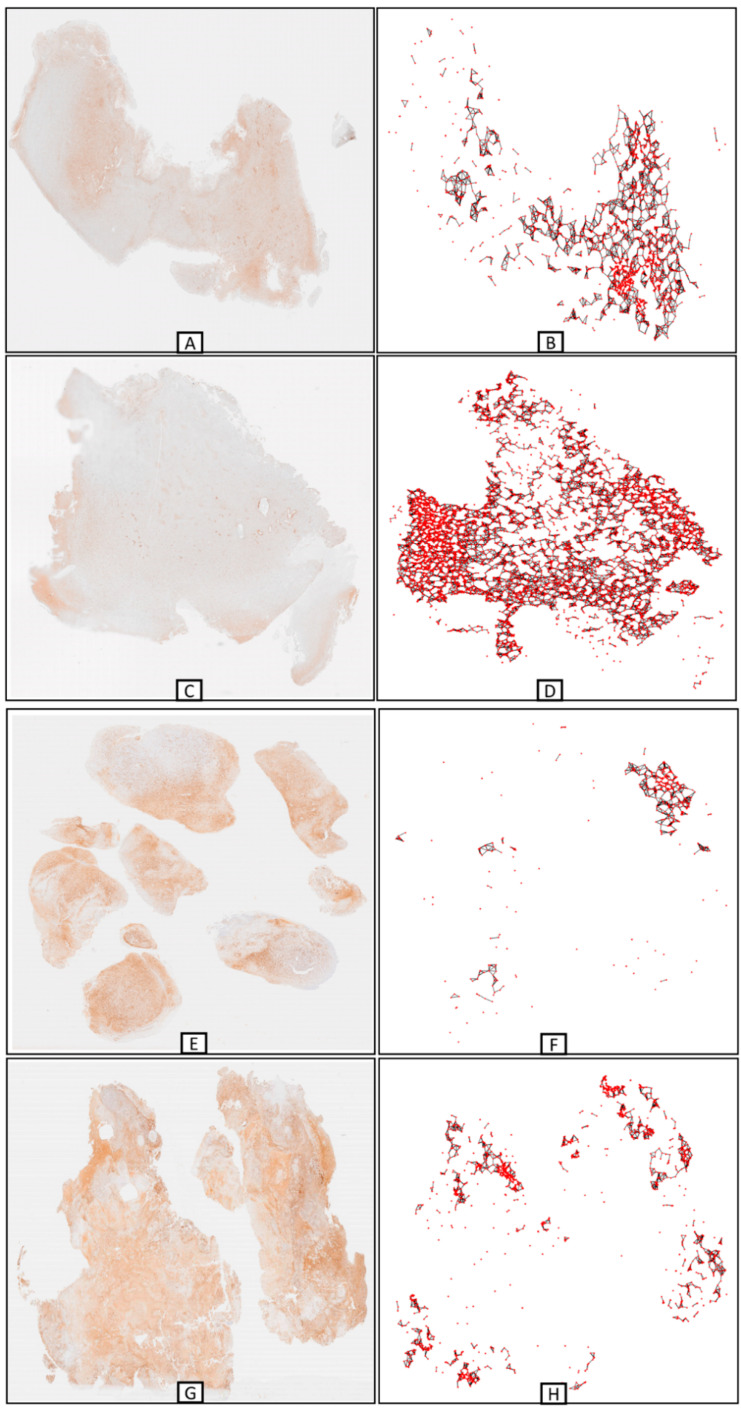
Graphs constructed from four sample WSIs on the basis of CD276+ GSCs detected by the BCNN (PathoFusion). (**A**–**D**) two cases with short survival and their corresponding graphs, (**E**–**H**) two cases with long survival and their corresponding much less complex graphs.

Each WSI yields a different number of nodes which when connected through edges result in one or more graphs of varying complexity. The number of nodes in our graphs that are based on the CD276 dataset of each WSI ranged from less than 100 to 15,000 per WSI. Sometimes biopsies can consist of multiple small tissue fragments. It should be noted therefore, that our proposed method requires the size of the fragments that are selected for analysis to be larger than the maximum ED employed. The nodes in the graph network represent the digital patches that contain CD276 immunoreactive cells, and the edges reflect the connection between the patches. Figure 4 depicts examples of graphs (right column) and the corresponding immunohistochemically labelled (CD276) WSI (left column) they were derived from. We found that survival correlated not only with the number of CD276 positive GSCs but also with greater graph complexity representing each GSC community contained in the WSI. Graphs were visualized with the help of networkx, a python network module.

### 3.3. Performance Evaluation of the abGCN Model Used by PathoGraph

For our experiment to validate the graph construction method independent of PathoFusion, we used the publicly available TCGA glioblastoma (GBM) dataset [28]. This dataset does not contain CD276 immunohistochemical stains but instead contains H&E histology. Accordingly, we have used the HoverNet model [31] for the simple extraction (segmentation) of nuclei from H&E-stained histology images. Subsequently, the corresponding node features were extracted using a ResNet34 pre-trained torch model. Finally, the graph was built using our graph construction method. The resulting graphs were used to train and validate our abGCN model to assess its performance.

For the CD276 dataset, at the graph construction stage, image augmentation including rotation, contrast, and sharpness adjustments was randomly applied to cell patches before they were used to generate the graphs. The actual generated graphs were therefore diversified and expanded greatly ($n^{2}$ times) compared to conventional methods (n times), where n is the number of augmentation types.

We used five-fold cross-validation to assess the performance of our abGCN model for predicting system outcome (patient survival in our example). We split each of the WSI datasets into a training set (80%) (used 25% of them as the validation set) and a testing set (20%). The model was then trained to perform dichotomous categorization (short and long survival, respectively) and to calculate attention scores (risk values) using the training set. Our abGCN model was trained like other state-of-the-art methods for comparison using the Adam optimizer with a weight decay factor of 0.0001. Training was conducted for 100 epochs at a learning rate of 0.001. The predicted risk associated with the input graph is computed by the last fully connected layer. These predicted risks of the input graphs are then fed into the model’s Cox proportional hazards regression loss function to minimize those risks and improve the model’s accuracy and thus ability to predict system outcome (survival). The model uses back-propagation to update the parameters in order to minimize the loss.

Table 1 shows the results of the evaluation of our abGCN model compared to those of existing multiple-instance or contextual feature learning models. Our model outperformed other models with a C-index of approximately 83% and 70% and an accuracy of 85% and 83%, when classifying patient survival (short or long survival) using CD276 dataset and the TCGA GBM dataset, respectively.

### 3.4. Visualization of Attention Scores

Our abGCN model has one attention gate which consists of three layers. It differentially assigns weights to the cells of interest in each of the WSI depending on their feature content and thus highlights their relative importance for the final evaluation. The proposed method produces a rich and interpretable map showing the contribution of each cell and its surroundings to the outcome parameter. The outcome parameter provides an explanation for the patterns found by the deep learning algorithm.

**Figure 5:**
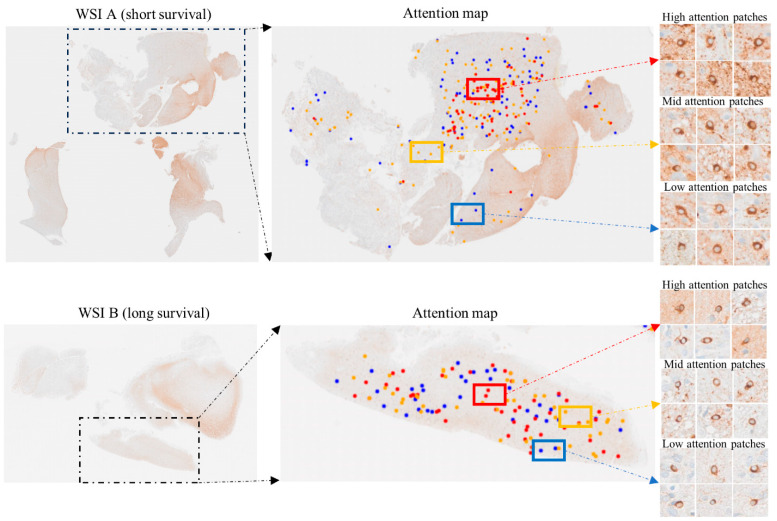
Attention scores allow the localization of tissue regions of special interest. This is illustrated using two sample areas from WSIs of cases with different survival outcomes (short and long survival, respectively). The colour coding pinpoints clusters of GSCs showing the highest CD276 expression (red).

In Figure 5, we show a visualisation of the attention scores of the cells that were detected in the selected regions. We utilise colour labels with, e.g., red representing a high weight (score; please see below). This aids our understanding of which elements of the graph influenced the model’s predictions most. Figure 5 shows two WSIs with different survival outcomes (short and longer term, respectively). The attention score can be used as a guide on where to focus on the tissue section to find meaningful differences between regions. For instance, they can be used to find areas with especially aggressive tumour growth.

After calculating the attention scores over the entire dataset of each WSI, we group the attention scores into high (>90%, red), medium (60–90%, orange) and low (<60%, blue). This is followed by selecting a set of patches from each group based on their attention score. Knowledge of the attention scores allows a contextual analysis of these patches, i.e., how the different groups relate to regions of interest in the WSI that are defined by pathological features which are recognizable when using other modalities on adjacent sections.

## 4. Discussion

Our abGCN captures a distinct property of the CD276 high-expressing (CD276^high^) malignant glioma cell population (GSCs) in each slide in the form of graphs that are representative of the respective biopsy/case and based on global information from the WSI. Importantly, more complex graphs are found to correlate with shorter survival. One biological interpretation could be that the more complex graphs represent more aggressively growing clones of GSCs. The underlying information is not readily accessibleto a human observer in most cases illustrating the power of the abGCN. In addition, the attention scores allow the localization of such cell communities within the tissue sections. These findings are intriguing and may have implications for histopathological prognostication and treatment decisions.

Recent studies have demonstrated that elevated expression of CD276 may have a role in inducing the epithelial–mesenchymal transition (EMT) [35]. Jiang and colleagues [36] have shown that upregulated CD276 in primary colon adenocarcinoma can enhance the expression of CD133 and CD44 associated with the EMT in colorectal cancer. CD133 and CD44 are cell membrane markers frequently used to identify cancer stem cells and colorectal tumour-initiating cells. Using digital spatial profiling, Petterson et al. [37] have observed elevated expression of B7-H3 (CD276) and CD44 in areas of hypoxia in GBM tissue. In GBM, B7-H3 is also prominently expressed by pericytes and macrophages in GBM tumour tissue [38]. It is worth noting that elevated B7-H3 expression and increased infiltration of macrophages in colorectal carcinoma have been negatively correlated with patient survival [39]. It has also been suggested that upregulated CD276 expression promotes macrophage differentiation towards an anti-inflammatory phenotype [39]. The elevated expression of B7-H3 by microglia and macrophages in glioblastoma may facilitate immune evasion [37].

Certain existing deep learning methods, for instance CNNs, have been proposed for survival analyses using WSIs and have shown promise when trying to understand disease progression and predicting patient outcome [40,41]. Mobadersany et al. [40] proposed a method to process 1024 × 1024 image ROIs using CNN and to classify survival outcomes using a loss of function tailored to survival analysis, ensuring that feature extraction and survival analysis are optimized together. Zhu et al. [41] have developed a two-step- approach for WSI-level survival outcome prediction, in which patches are clustered using a K-Means clustering method with the results then used as input for a CNN. However, when dealing with relation-aware representations, CNN models are inefficient because they suffer from a limited ability to capture global contextual information and thus model tissue composition comprehensively. In tissue diagnosis, the phenotypic characteristics and topological distribution of cellular and histological features are of critical importance. Consequently, GNNs have attracted considerable interest as they capture intra- and inter-entity level interactions [18] and are able to record geometrical alongside topological properties, thus allowing cell-level information and overall tissue micro-architecture to be modelled simultaneously. GNNs have already been used for ROI classification [42], the detection of malignant growth and tumour invasiveness [43], as well as survival-analysis on whole slide pathological images [34].

Jaume et al. [19] have proposed metrics to evaluate three types of graph explainers to understand cell graph representations for breast cancer subtyping. After the cell graph was constructed using ROIs in the histology image, a GNN model was then used to map the generated graph to a corresponding class label. Then, they employed a post hoc graph explainer and a set of metrics was used to assess the generated explanations. Also, a cell-graph explainer was used in breast cancer subtyping [44] to eliminate redundant and non-informative graph nodes and edges while retaining tumour cell nuclei relevant for cancer diagnosis. Anand et al. [43] developed a framework named Histograph for breast cancer WSI classification. The framework utilizes a pre-trained model to extract features around the nuclei centroids and represents nuclei as graph vertices and their inter-nuclear distances as graph edges. Sureka et al. [21] have also employed a graph-based approach to model histopathology images as graphs of nuclei to classify breast cancer biopsies. They used a U-Net for nuclei detection, constructed a graph based on nuclei proximity, and developed a GCN framework with an attention mechanism and node occlusion to emphasize the relative contribution of each nucleus and its neighbourhood in the WSI in the classification process. Zhou et al. [45] developed a GCN for colorectal cancer classification using a graph representation of WSI. The graph nodes are represented by nuclei while the edges between these nodes are built according to node similarity.

## 5. Conclusions

We have developed a graph-based AI model for the detection and interpretation of immunohistochemical labelling results at the cellular and WSI level. In the case of CD276 when used as a marker of GSCs in malignant glioma, the method allowed us to predict system outcome on the basis of the graphs and attention scores that were generated. Specifically, our PathoGraph framework containing an abGCN was able to classify survival outcome and the attention scores allowed pinpointing of WSI areas of particular diagnostic interest. Therefore, our graph visualization method is able to uncover interpretable information that is largely hidden to a human observer. Employing a GCN with attention heads resulted in improved performance. While our current model shows promising results in classifying scanned slide images based on survival outcomes, we acknowledge that a larger dataset would be advantageous. A more extensive dataset would not only enhance the robustness of our model but also enable more sophisticated analyses and potentially improve its predictive capabilities. We are actively working towards building a larger dataset to further refine and validate the performance of our model. Finally, we would like to point out the potential generalizability of the new method. It might be used for screening for novel biomarkers for example when different system outcomes and input data sets are combined.

## Figures and Tables

**Table 1:** Performance comparison of different deep learning models.

Model	C-Index		Accuracy	
	TCGA	CD276	TCGA	CD276
MIL (Deep Sets) [32]	0.64	0.63	0.77	0.79
Attention MIL [33]	0.68	0.59	0.80	0.78
DeepGraphConv [34]	0.67	0.56	0.80	0.73
abGCN (this model)	0.70	0.83	0.83	0.85

**Figure A1:**
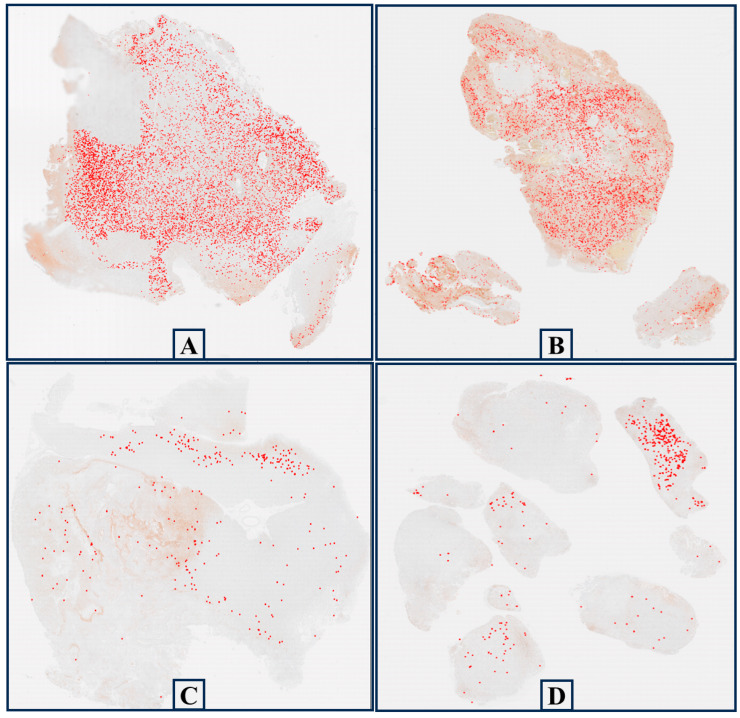
(**A**,**B**) Two cases with short survival, and (**C**,**D**) two cases with long survival. The red colour shows the detected CD276 GSCs.

## Data Availability

Sample datasets for image patches are openly available in https://github.com/guoqingbao/Pathofusion/tree/master/data, accessed on 15 December 2023.
